# Reports on the Hospitals of the United Kingdom

**Published:** 1909-12-18

**Authors:** Henry Burdett


### REPORTS

ON THE

### Hospitals of the United Kingdom.

By SIR HENRY BURDETT, K.C.B., K.C.V.O.

### XIV.—NOTTINGHAM GENERAL HOSPITAL.

### The Right Spirit.

When we inspected the hospital in 1892 we bore testimony to the cheerful spirit in which the staff did their work, and to the fact that the medical and surgical results were so good as to afford practical evidence of the excellence of the nursing and of the control exercised by the resident and medical staff and by the Matron. The same spirit dominates the whole of this hospital to-day, and all that we said of the resident staff of 17 years ago may be repeated with emphasis and truth of the ladies and gentlemen who constitute the resident staff to-day. We spent several hours in, and made a complete and detailed inspection of, the entire establishment. It was remarkable and pleasant to find evidence almost everywhere that thoroughness and devotion to duty were the keynotes which have made this hospital so splendidly efficient in all its departments. Although the hospital does not possess a modern and up-to-date nurses' home, the quarters assigned to individual nurses are, on the whole, satisfactory, and the Matron has just cause to be proud of the spacious cubicles which she has secured for her wardmaids. The night nurses are isolated, and in this hospital it should be possible for them to obtain plenty of sound sleep in undisturbed quietude.

### Well Done, Nottingham.

We pointed out in The Hospital of August 13, 1892, that the support then extended to the hospital by the people of Nottingham was most inadequate, and compared very unfavourably with the gifts to other similar hospitals at Birmingham and elsewhere. The contributions of the people at Nottingham at that time, including receipts from the anniversary and ball, were under £6,000. In 1908 the sum received from these sources exceeded £11,500. We made an urgent appeal for an increased income for the hospital of £3,000 a year, which was promptly forthcoming, and is now a fixed and regular contribution. The annual subscriptions have increased over 50 per cent., or from £2,072 to £3,178, and the working classes have increased their gifts from £1,366 to £5,695 a year, that is, by over 300 per cent. This splendid growth in the revenue derived from individual givers is not maintained in the church and chapel collections, which are practically stationary—*i.e.* £1,079 in 1891 and £1,159 in 1908. The care in the management of the affairs of the hospital is testified to by the fact that the dividends, etc., derived from invested funds have increased from £1,244 to £2,040. Unfortunately the ordinary expenses have increased from £8,000 to £15,417. Still, the revenue of the hospital shows an excellent vitality, and would seem to indicate that the people of Nottingham now take a real interest in their hospital, and are proud of it, as they have good reason to be. That is evidently true of the working classes, whose representatives on the committee have done excellent service, whilst their annual contributions to the hospital do them great honour, and should stimulate other sections of the community to awake and give something to the hospital fund every year.

### Diets and the Service of Meals.

We think a greater supervision should be exercised over the distribution of food from the kitchens to the wards. We saw hot diets placed in cold tins before they had been heated by hot water. This fact points to the certainty that some patients are liable to have tepid or cold food served to them in the wards. This is not right, and someone ought to inspect the dinner tins in the kitchen every day, and to see that they are properly heated before any food is placed in them for despatch to the wards. On examining the diet sheet for the day we found that only one patient out of upwards of 200 had a full diet. This would seem to point to an unusually high average of serious cases, for we cannot suppose for one minute that the patients are not liberally treated in respect of food.

### A Good Record.

This hospital, though several parts of it are old, is one that should be visited by all who wish to see what good administration can produce under difficulties not always easy to master. Before our inspection we felt it probable that this hospital would not compare favourably, on account of the age of some of its buildings, with some of the more recently built institutions in neighbouring counties. We found, however, that it stood out prominently amongst them all on account of the spirit, intelligence, and zeal which now characterised the work of every member of its staff. Seventeen years ago, when we last inspected it, we found the staff excellent and the wards and departments exceptionally well kept. The same may be said of the hospital to-day after its reconstruction, for it is an up-to-date and in many respects a good type of a modern hospital. We are glad to note, as is but fitting, that our appeal for a larger income made seventeen years ago has been answered to the full, and that the business side of the administration is kept well to the front, as the adoption of the uniform system of accounts proves. It seems to us, however, having regard to the size and importance of this hospital, that an inquiry into the cost of the secretariat, which is, strange to say, too small in our judgment, should be entered into, if it has not already taken place. No money is better laid out than that paid to hospital workers, for every man and woman who devotes his or her life to the sick should be taken special care of by the committees of these institutions.

We have had the pleasure to give much praise in this report, but this is bare justice, bearing in mind as we do what this hospital once was and what efficient workers have now made it.

### XV.—SOME COTTAGE HOSPITALS.

### DORKING COTTAGE HOSPITAL.

This hospital was established in 1871. It would not be fair to judge it by present standards of construction. The wards have no cross ventilation, and the sculleries, lavatories, and also the heating arrangements leave something to desire. We found the hospital perfectly clean throughout, but it seemed to us that the wards did not present that orderly appearance which we expect to find. The women's ward, especially, was ill ventilated, and had a disorderly appearance, largely due to the presence of too many rather clumsy, but evidently useful, invalid bed-tables. There appears to be an unusually large number of out-patients at this hospital, and it is the practice, we understand, for the staff to send in their private patients requiring minor operations and to treat them in the hospital. The operation theatre is exceptionally good and well kept. The out-patient accommodation is a special feature of this cottage hospital. The larder badly needs ventilation, and it is desirable, especially in view of the date at which the wards were built, that open-air shelters or large verandahs at least 10 feet wide, should be provided, so that patients may sleep in the open air. Baths, of which there are three, are a special feature of this hospital. They are good baths and splendidly kept. The matron, Miss Turner, a London Hospital nurse, is deeply interested in her work, and has the assistance of one charge nurse and two probationers. We congratulate the Honorary Secretary upon the fact that Dorking is one of the very few cottage hospitals which keep their accounts on the uniform system. The expenditure exceeds the ordinary income by nearly £300, and we could wish that this useful hospital was better supported throughout the district which it serves so well.

**The Coventry and Warwickshire Hospital** will be reported on next week.

